# Use of a modular hip dual-mobility articulation in patients with high risk of dislocation: a relatively small-sized acetabulum in Asian patients may limit its use

**DOI:** 10.1186/s42836-020-00066-0

**Published:** 2021-05-03

**Authors:** Ping Keung Chan, Sum Lik Cheung, Kar Hei Lam, Wing Chiu Fung, Vincent Wai Kwan Chan, Amy Cheung, Man Hong Cheung, Henry Fu, Chun Hoi Yan, Kwong Yuen Chiu

**Affiliations:** Department of Orthopaedics & Traumatology, Queen Mary Hospital, The University of Hong Kong, 102 Pok Fu Lam Road, Hong Kong, Hong Kong SAR

**Keywords:** Dislocation, Dual mobility, Osteoarthritis, Total hip arthroplasty

## Abstract

**Background:**

Dual-mobility hip component is widely used in Europe and North America, because it effectively reduces hip dislocation in primary and revision total hip arthroplasties. However, reports were limited on the use of dual-mobility articulation in Asian populations.

**Purpose:**

The aim of this retrospective study was to review the use of modular dual-mobility hip articulation in Asian patients with the high risk factor for hip dislocation. We also discussed the potential concern on the use of dual-mobility articulation in Asian patients.

**Methods:**

From Jan 2018 to June 2019, 17 patients were included in this study. The mean age of the patients was (73.8 ± 9.5) years (range: 57–88 years). The mean size of acetabular cup and modular DM liner were (49.5 ± 3.4) mm (range, 46–58 mm) and (40.7 ± 3.4) mm (range, 38–48 mm), respectively. The mean follow-up period was (15.8 ± 3.9) months (range, 11–24 months). The primary outcome was the rate of hip dislocation. The secondary outcomes included the Harris Hip Score. Differences were considered statistically significant at *p* < 0.05.

**Results:**

Hip dislocation, loosening, peri-prosthetic fractures, or intra-prosthetic dislocation was not found in the series. The mean preoperative and postoperative Harris Hip Scores were 42.2 ± 17.2 (range, 15–80) and 74.7 ± 13.5 (range, 52–97), respectively, giving a mean improvement of 32.5 ± 17.2 (range, 4–72). The improvement was statistically significant (*p* < 0.05).

**Conclusions:**

In Asian patients with high risk of hip dislocation, the use of modular dual-mobility hip component produces promising outcomes without hip dislocation, but the relatively small-sized acetabulum may limit it widespread application in other populations worldwide.

**Trial registration:**

HKUCTR-2913.

## Background

Apart from aseptic loosening and infection, another common cause of revision after primary total hip arthroplasty (THA) was instability/dislocation, accounting for 23% of all THAs, according to an epidemiological study involving 51,345 revision THAs performed in the United States [1].

In 1974, Gilles Bousquet and Andre Rambert first proposed the dual-mobility (DM) concept. The DM concept combines the principles of Charnley’s low-friction arthroplasty with the McKee-Farrar concept of increasing femoral head-to-neck ratio to maximize hip stability [2, 3]. In a DM acetabular cup, there is a mobile polyethylene liner interposed as an additional bearing between the prosthetic head and the acetabular shell [4]. Several meta-analyses and systematic reviews showed the benefit of DM articulation in reducing postoperative dislocation in THA [5–7]. In a recent comparison study, Romagnoli et al. [8] showed the risk ratio of DM bearing group was 0.16, against a higher risk ratio of the conventional bearing group.

DM articulation was used worldwide for more than 20 years. In the early years, DM articulation was mainly used in the European countries [5]. In 2009, DM design was approved by the United States Food and Drug Administration, and was widely used in North America thereafter due to an increase in availability of the modern DM design. The American Joint Replacement Registry [9] reported an increasing trend of using DM articulation in both primary and revision THAs. The percentages of DM system used in the primary THA increased from 3% in 2012 to 7% in 2018, while the DM system employed in revision THAs rose from 11% to 16% [9]. However, to the best of our knowledge, there were only five English-language reports discussing the use of DM articulation in Asian patients [10–14].

Hip dislocation is one of the major complications of THA. The reported high risk factors include neurological disabilities (cognitive, motor, or psychiatric disorders), a history of spinal diseases (lumbar stenosis and spinal fusion), etc. [15]. In order to decrease the incidence of hip dislocation, DM articulation was suggested for patients with the risk factors.

This retrospective study aimed to review the use of modular DM hip articulation in Asian patients with the high risk factor of hip dislocation. We also discussed the potential concerns over the use of the implant in Asian populations.

## Patients and methods

This retrospective review was approved by the institutional review board (IRB Reference Number UW 19–848). From January 2018 to June 2019, 17 consecutive patients underwent DM THAs in our hospital. The patients were invited to participate in the study after informed consent had been obtained. Our eligibility criteria were high risks of hip dislocation after THA, including abductor deficiencies, a fractured neck of femur, neuromuscular diseases, neurological disabilities (cognitive, motor, or psychiatric disorders), a previous hip surgery (i.e. revision, conversion surgeries), and spinal pathologies (lumbar stenosis, spinal fusion, discectomy, scoliosis, degenerative disc disease) [16–22]. Patients who refused to participate in the study were excluded. All operations were preformed by the same orthopaedic surgeon (PKC), who had a post-fellowship training in arthroplasty for more than 10 years.

### Surgical Technique

**Fig. 1:**
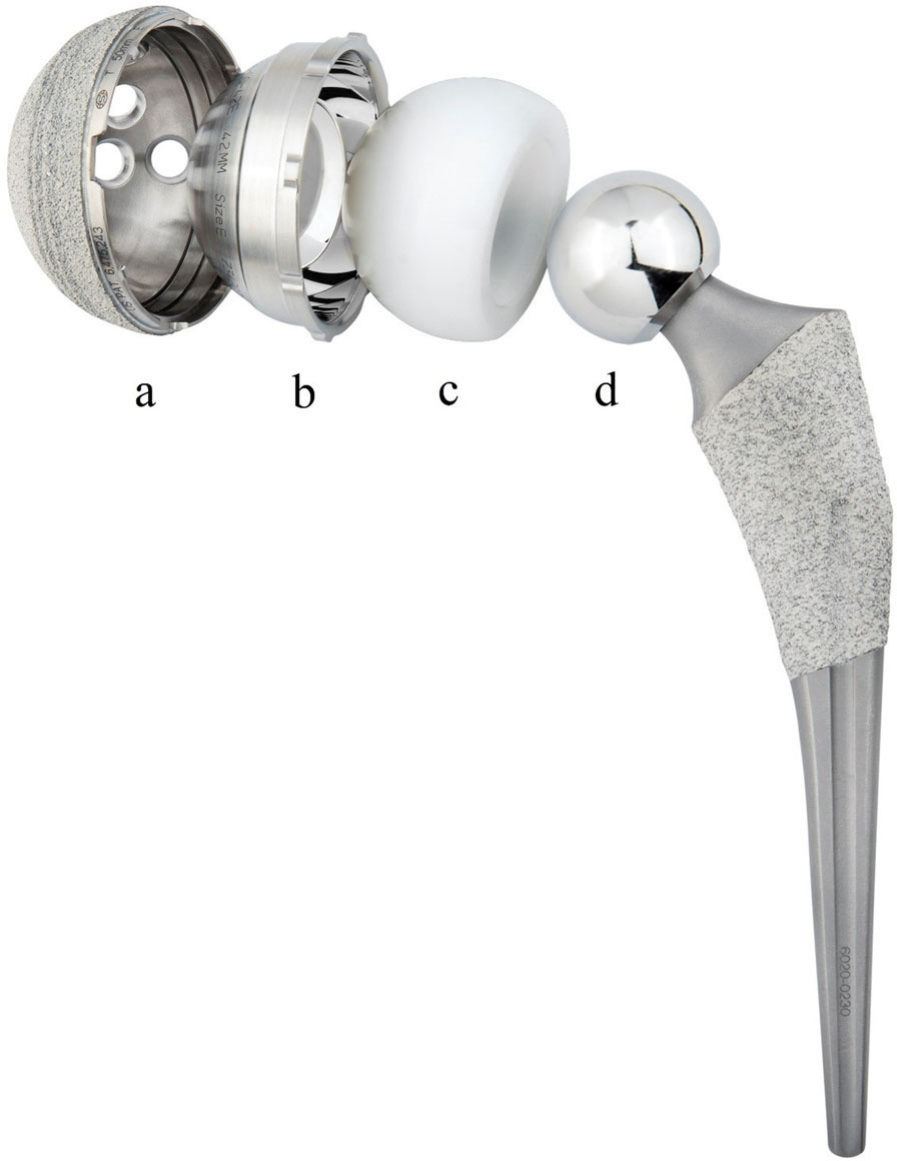
The modular dual mobility (DM) used in the current study (**a**, acetabular cup; **b**, modular DM liner; **c**, polyethylene insert; **d**, femoral head)

The patient was placed in the lateral decubitus position on an arthroplasty specific operating table. Under spinal or general anaesthesia, the operation was performed through the posterior approach. The short external rotators and the posterior capsule were exposed and tagged (to facilitate later repair after the prosthesis had been implanted to enhance hip stability) [23]. The acetabular cup used in this study was Trident PSL Shell (modular DM, Stryker, Mahwah, New Jersey, USA) (Fig. 1). The acetabular cup was implanted with the press-fit technique according to the manufacturer’s recommendation. The acetabular component orientation was adjusted according to the Lewinnek safe zone (inclination 30–50°; anteversion 5–25°) [24]. The cup alignment was aimed at 20° of anteversion and 40° of inclination. A cup implanted outside of the Lewinnek safe zone was considered to be in a suboptimal position, which was improved with 2 to 3 supplementary trans-acetabular screws to enhance its mechanical stability. In primary THA, a primary cemented femoral stem (Exeter, Stryker) was used for patients with Dorr Type C stove-pipe canal [25], and a cementless femoral stem (Accolade II or Restoration HA, Stryker) was used for patients with other femoral geometry. In revision THA, the original femoral stem with proper orientation was retained if there was no sign of loosening. Otherwise, the stem was revised with a revision femoral stem (Restoration HA, Stryker). In conversion THA (failed hemi-arthroplasty or failed fixation due to hip fracture), a primary or revision femoral stem was selected. Alternatively, the original femoral stem could be retained only if bone stock was adequate, stability sufficient, and orientation correct. Attention was turned to optimizing the leg length restoration, and intraoperative range of motion, and then stability of the implant were assessed. The posterior capsule and short external rotators were repaired, and the incision was closed in layers.

### Postoperative management

After surgery, the patient stayed in bed, with a hip abduction pillow placed between the two legs. The patient was put on the standardized rehabilitation protocol and trained by the same physiotherapist. Functional training was given by the same occupational therapist. The patient was advised to take the standard hip precautions (avoiding flexion > 90°; adduction > 10°; internal rotation > 10°; crossing of legs; picking up low objects; sitting in low chairs; or prone sleeping position) for 6 weeks. After discharged, the patient was followed up in the out-patient clinic with repeated X-ray examinations after 6, 3, 6 months, and one year.

### Outcome evaluation

Patients’ surgical notes, prosthesis details, medical records, and X-rays were reviewed. The primary outcome was the dislocation rate of THA. The secondary outcomes included the Harris Hip Score (HHS). Complications included prosthetic loosening, peri-prosthetic fractures, intra-prosthetic dislocation, among others. The collected data were analyzed with the *t*-test. Differences were considered statistically significant at *p* < 0.05.

## Results

**Fig. 2:**
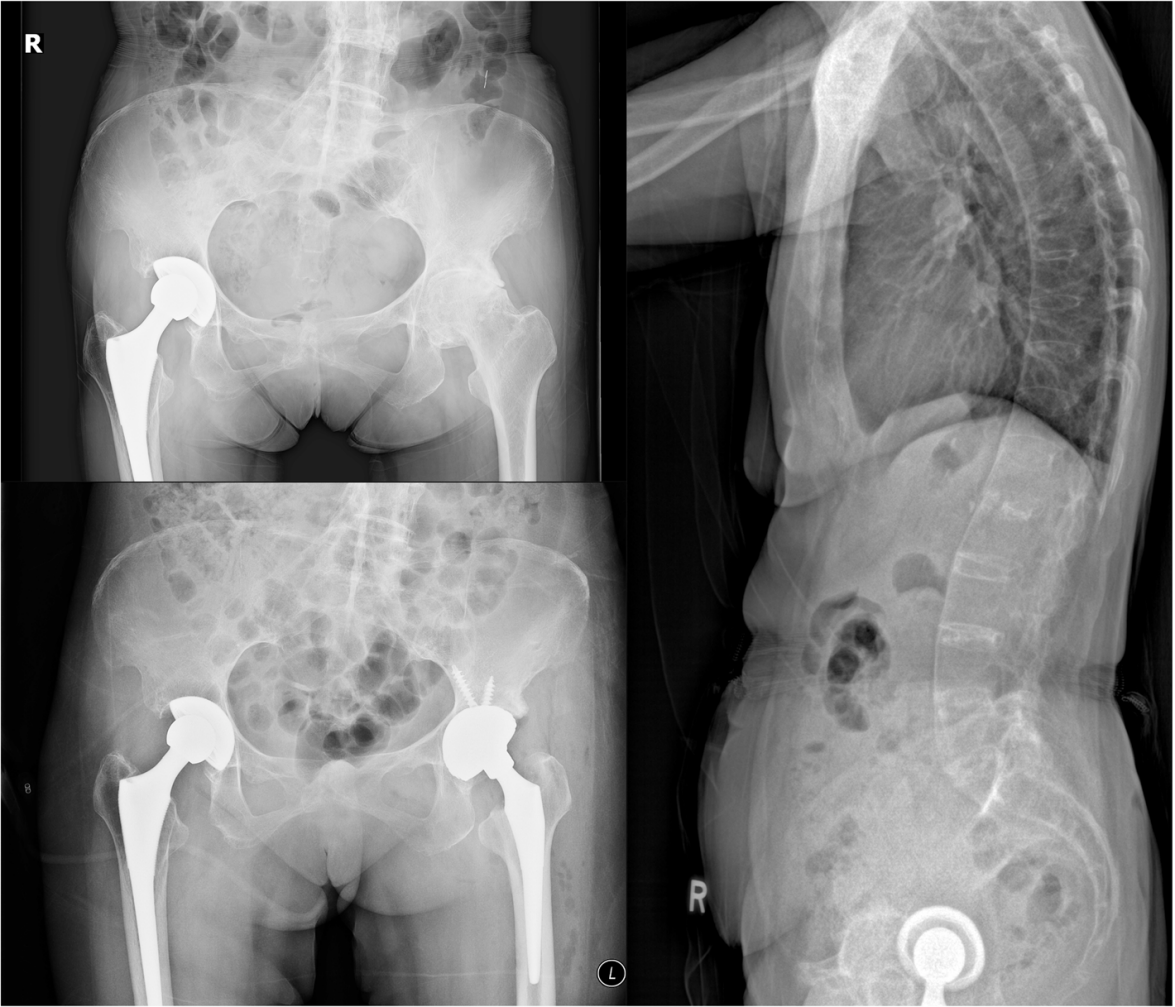
Case No. 6 in Table 1. **a** Lateral X-ray showing whole spine spontaneous fusion because of inflammatory arthritis. **b** Osteoarthritis on the left hip. **c** DM THA on the left hip with spinopelvic imbalance

**Fig. 3:**
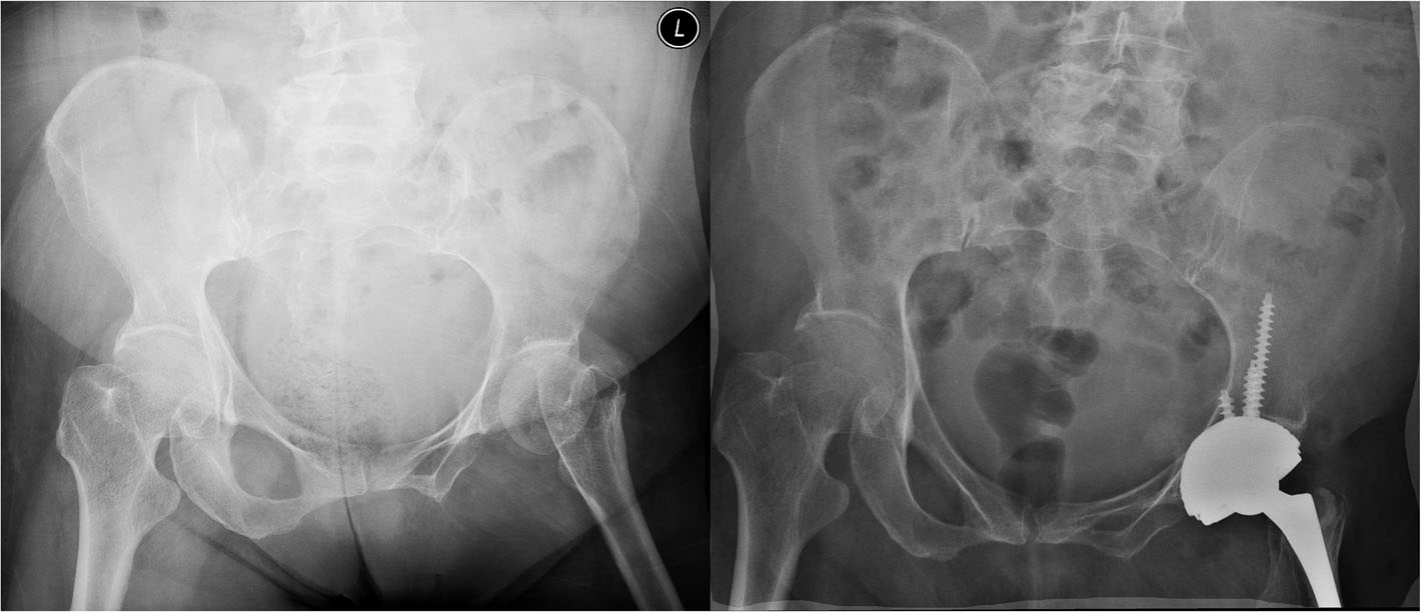
Case No. 7 in Table 1. **a** The left hip had a fractured neck of femur with abductor deficiency due to poliomyelitis. **b** DM total hip arthroplasty on the left hip

A total of 17 patients (male:female = 2:15) were included in this study, and no patient was excluded. The mean age of the patients was (73.8 ± 9.5) years (range, 57–88 years). There were 9 primary, 6 conversion, and 2 revision THAs. Patients’ characteristics and surgical details are shown in Table 1. The reasons for choosing DM bearings included abductor deficiency (*n* = 3), spinal pathologies (*n* = 6), fractured neck of femur (*n* = 5), previous hip surgeries (*n* = 8), and neurological disabilities (*n* = 1). Six patients had two risk factors of dislocation, and 11 patients had one risk factor. Two typical cases are shown in Figs. 2 and 3.

**Table 1:** Details of the patient’s demographics, prosthesis and operative information, and outcomes

Case No.	Age	Sex	OT Date	Diagnosis	Laterality	Type	Risk factors for THA dislocation	Acetabular Cup Size(mm)	MDM Liner Size(mm)	Hip Ball Size(mm)	Femoral Stem	Preop HHS	Postop HHS	Change in HHS	Follow up duration (months)
1	82	F	6/6/2019	Infected THA	L	Revision	D	46	38	22.2	Restoration HA Stem	62	73	11	11
2	59	M	25/4/2019	OA hip 2nd to ankylosing spondylitis	R	Primary	B	54	46	28	Accolade II	41	85	44	13
3	74	F	14/3/2019	#NOF, Poliomyelitis	L	Primary	A, C	52	42	28	Exeter	43	81	39	14
4	81	F	7/3/2019	OA hip 2nd to rheumatoid arthritis	L	Primary	B	48	38	22.2	Exeter	41	81	40	14
5	87	F	22/2/2019	Failed hip fracture fixation	L	Conversion	D	48	38	22.2	Restoration HA Stem	25	54	29	12
6	68	F	14/2/2019	OA hip 2nd to psoriatic arthritis	L	Primary	B	46	38	22.2	Accolade II	31	75	44	12
7	71	F	1/12/2018	#NOF	L	Primary	A, C	48	38	22.2	Exeter	8	80	72	17
8	59	F	20/12/2018	OA hip 2nd to hip dysplasia	L	Primary	B, E	50	42	28	Exeter	58	97	39	12
9	72	M	8/11/2018	Post traumatic AVN	L	Conversion	D	54	46	28	Accolade II	39	86	47	13
10	57	F	6/9/2018	#NOF	L	Primary	C	50	48	28	Accolade II	5	67	62	16
11	71	F	16/8/2018	OA hip 2nd to TB	R	Primary	A, D	46	38	22.2	Accolade II	33	60	27	20
12	88	F	1/8/2018	Failed hip fracture fixation	R	Conversion	D	48	38	22.2	Restoration HA Stem	32	54	22	14
13	75	F	16/7/2018	Recurrent dislocation of hemiarthroplasty	L	Conversion	D	50	42	28	Original stem retained	32	52	20	18
14	69	F	25/5/2018	Acetabular cup loosening	L	Revision	B, D	58	42	28	Original stem retained	70	74	4	21
15	78	F	11/5/2018	Post traumatic AVN	L	Conversion	C	50	42	28	Accolade II	30.5	90	59.5	24
16	84	F	1/3/2018	Neglected #NOF	R	Primary	B, C	48	38	22.2	Accolade II	15	74	59	14
17	79	F	11/1/2018	Loosening of hemiarthroplasty	R	Conversion	D	46	38	22.2	Restoration HA Stem	80	84	4	23
	Mean Age	M: F			L: R	Primary: Conversion: Revision	No. of Patients	Mean size	Mean size	22.2mm: 28 mm		Mean (n = 15)	Mean (n = 15)	Mean (n = 15)	Mean
	73.8 ± 9.5	2: 15			12: 5	9: 6: 2	A = 3 B = 6 C = 5 D = 8 E = 1	49.5 ± 3.36	40.7 ± 3.39	9:8		42.2 ± 17.2	74.7 ± 13.5	32.5 ± 17.19	15.8 ± 3.9

*AVN Avascular necrosis, *#NOF *Fractured neck of femur, *TB* Tuberculosis

*A = Abductors deficiency, B = Spinal pathology, C = Fractured neck of femur, D = Previous hip surgeries, E = Neurological disabilities

The mean size of acetabular cup and modular DM liner was (49.5 ± 3.4) mm (range, 46–58 mm) and (40.7 ± 3.4) mm (range, 38–48 mm), respectively. The mean follow-up period lasted (15.8 ± 3.9) months (range, 11–24 months). Hip dislocation, loosening, peri-prosthetic fractures, or intra-prosthetic dislocation was not found in our series. No revision THA was required. We excluded two patients with hip fractures, because their preoperative HHSs were not available. For other 15 patients, the mean preoperative and postoperative HHSs were 42.2 ± 17.2 (range, 15–80) and 74.7 ± 13.5 (range, 52–97) respectively, achieving a mean improvement of 32.5 ± 17.2 (range, 4–72). The improvement was statistically significant as revealed by paired *t*-test (*p* < 0.001).

## Discussion

A DM implant consists of two articulations. The first articulation is a small inner femoral head fitting the inside of a large hemispherical polyethylene insert. The second articulation is the polyethylene insert articulating with the outer acetabular shell. The inner articulation is responsible for the primary movement of the prosthetic joint, while the outer articulation moves only at the extreme range of movement [26]. DM improves stability and increases range of motion of the hip joint, by means of the increased the head-to-neck ratio, a large head size, and a great jump distance [27]. DM articulation is also associated with low dislocation rate and revision rate. Reina et al. [28] reviewed 12 comparison studies published between 2015 and 2018, in which 1132 DM THAs and 1583 conventional THAs were included. With primary THA, the overall dislocation rate of DM implant was 1%, as compared to 7% of the conventional implants. In revision THA, the overall dislocation rate of DM was 2%, in comparison with 7% of the conventional implants [28]. The modular dual mobility (Stryker, Mahwah, New Jersey) (Fig. 1) is the only DM designed implant in our region.

### DM hip components in Asia

Mounting evidence has shown the benefits of DM components, but in Asian countries there are only 5 English-language publications on the use of DM articulation, including 3 in Japan [10–12] and 2 in Korea [13, 14]. Since 2013, Homma et al. [10] first performed DM THA in 121 patients aged over 70 years, and in patients between 65 and 69 years old who had a high risk of hip dislocation or short life expectancy. They concluded that DM THA is an effective technique in managing patients with femoral neck fracture, and in preventing the high risks of complications [12]. Kim et al. [13] conducted a comparison study between the DM THA and bipolar hemi-arthroplasty for treating displaced femoral neck fractures, and found that the former resulted in a better hip function with lower mortality or dislocation rate.

### Acetabular cup size in Asian population

In a dry cadaveric study, Hoaglund and Low compared the sizes of femoral heads in the Caucasian and Hong Kong Chinese populations [29]. Adding an average cartilage thickness of 3 mm to the femoral head, they found that the femoral head diameter of the Hong Kong Chinese population (43 mm in women; 48 mm in men) was smaller than that of the Caucasian population (46 mm in women; 49 mm in men). Lee et al. [30] also reviewed 945 Malay patients’ femoral heads, including ethnicity of Malay, Chinese, and Indian origins. By measuring the femoral heads taken from the patients who underwent hemi-arthroplasty, they found that the mean diameter of femoral head was (44.2 ± 3.0) mm in Malay patients, (44.4 ± 3.3) mm in Indian patients, and (45.2 ± 3.1) mm in Chinese patients. Therefore, the size of femoral head, and hence the size of acetabulum in Asian populations was relatively smaller than that of the Caucasian populations. Since the smallest MD acetabular cup is 44 mm, it definitely limits its wide application and decreases the potential benefits for Asian populations [31].

### Selection of an appropriate modular DM acetabular cup

Dubin et al. [32] used modular DM articulation in 280 patients in a single hospital in the United States. In their cohort, the mean acetabular cup size was (53.1 ± 2.7) mm (range, 44–60 mm); and a 50-mm or 52-mm cup combined with a 42-mm modular DM liner was very common, accounting for 59% of patients in the cohort (Table 2). In our cohort, the mean acetabular cup size was (49.5 ± 3.4) mm (range, 46–58 mm). There was a statistically significant difference between the above two cohorts (*p* < 0.001). Approximately 53% of our patients used a 46-mm or 48-mm modular DM cup in combination with a 38-mm modular DM liner. In our hospital, most acetabular cup systems are suitable for the conventional THA, such as Pinnacle (Depuy Synthes), R3 (Smith & Nephew), and Continuum (Zimmer Biomet). The smallest cup size is 52 mm and it matches 36-mm femoral head ball. However, in Trident PSL Acetabular System (Stryker), there are smaller cup sizes (46 mm or 48 mm) that match a 36-mm femoral head ball if neutral polyethylene liner is used. Therefore, when a 46-mm or 48-mm cup is selected, a 38-mm mobile polyethylene has only a small increase in diameter compared to a 36-mm femoral head ball used in the conventional THA. Hence, the proposed benefit of DM articulation reduces hip dislocation by increasing primary arc range, and the jump distance was only marginally increased by using a 38-mm femoral head against a 36-mm femoral head used in the conventional THA. Other studies in Asian patients also showed the similar findings with the use of a relatively small cup in DM. Homma et al. [10, 11] also reported a median cup size of 50 mm (range: 46–58 mm) and 48 mm (range, 44–56 mm) in two studies. Hwang et al. [14] reported a mean cup diameter of (50.9 ± 3.0) mm (range, 44–62 mm).

**Table 2:** – Comparison of Acetabular Cup sizes for patients reported in Dublin’s study and in current study

Acetabular cup size (mm)	No. of patients in Dubin’s study in United States (%)	No. of patients in current study (%)
**44**	**1 (0.4%)**	**0 (0%)**
**46**	**1 (0.4%)**	**4 (23.5%)**
**48**	**1 (0.4%)**	**5 (29.4%)**
**50**	**54 (19.3%)**	**4 (23.5%)**
**52**	**110 (39.3%)**	**1 (5.9%)**
**54**	**49 (17.5%)**	**2 (11.8%)**
**56**	**39 (13.9%)**	**0 (0%)**
**58**	**17 (6.1%)**	**1 (5.9%)**
**60**	**8 (2.9%)**	**0 (0%)**
**62**	**0 (0%)**	**0 (0%)**
	**total = 280**	**total = 17**
**mean**	**cup size = 53.1 mm**	**cup size = 49.5 mm**
**median**	**cup size = 52 mm**	**cup size = 48 mm**

*p*-value < 0.001 (Independent samples *t*-test)

### Selection of an appropriate modular DM femoral Head

In the modular DM system, a 22.2-mm femoral head well matches a small-sized acetabular cup (Trident PSL cup), including 44-, 46-, and 48-mm cups. For an acetabular cup (Trident PSL cup) ≥ 50 mm, a 28-mm femoral head is selected. In our study, 53% of patients used 22.2-mm femoral head because of small-sized acetabular cup used whereas only 2% of patients received 22.2-mm femoral head among patients in Dubin et al’s study [32] (Table 3) (*p* < 0.001). Combes et al. [33] argued that intra-prosthetic dislocation was the only risk when a 22-mm head was used. This is of particular relevance to our patient group, because such head was used in 53% of our patients. Intra-prosthetic dislocation is the complication specific to DM articulation. It refers to a dislocation of polyethylene liner head from the inner femoral head as the consequence of the degeneration of the polyethylene retentive rim. The dislocated polyethylene liner is classically described as the C-shaped bubble displaced outside of the hip joint on X-ray. Upon review of 1960 primary DM THAs which were followed up for an average period of 14 years, Philippot et al. [34] found that the intra-prosthetic dislocation rate was 4%. Intra-prosthetic dislocation is one of the main concerns that limited DM usage, especially the early DM design. Levin et al. [35] reported that the incidence dropped to 0.3% with the use of the modern DM. Therefore, the modern DM is advised for small acetabulum in Asian patients.

**Table 3:** Diameter of femoral head used in MDM in Dublin’s study and in current study

Head Diameter (mm)	No. of patients in Dubin’s study (%)	No. of patients in current study (%)	*p*-value (Chi-square test)
**22.2**	**5 (1.8%)**	**9 (52.9%)**	**< 0.001**
**28**	**273 (98.2%)**	**8 (47.1%)**	

### Implant materials and design

With the first-generation DM bearings, the stainless-steel acetabular socket was coated with alumina and the inner surface was polished. The inner femoral head was made of metal, and the mobile layer was made of ultra-high molecular weight polyethylene [36]. The first-generation design was criticized by Blakeney et al. [37] for its undesirable outcomes, i.e., 3% of implant loosening, 2% of significant wear, and 5% of intra-prosthetic dislocation [34]. These complications were mainly the results of the poor cup fixation and premature wear of polyethylene layer, particularly at the retentive rim [37]. Since the early 2000s, there has been a remarkable improvement in terms of implant design and materials used. The modern DM bearings were coated with a bilayer of porous titanium, with or without hydroxyapatite, instead of alumina as the acetabular coating, to optimize bone fixation [38]. As a result, the decreased cup loosening renders the implant comparable to the fixed inserts used in the conventional THA [39]. Moreover, the highly cross-linked polyethylene (HXLPE) used with enhanced polyethylene rim durability and the additional retentive chamber have substantially improved the long-term survivorship of the modern implants [38, 40]. Together with a more polished and thinner femoral neck [38, 40], the modern design decreases the risk of intra-prosthetic dislocation and achieves a better retention mechanism of the polyethylene head [37]. The superiority of modern designs is supported by laboratory and clinical data. Netter et al. [41] tested the HXLPE DM implant and found it had excellent tolerance for third-body particles and good reduction in micro-separation. In 2013, Stryker Orthopaedics compared modern HXLPE DM implants and the first-generation implants, and found that the former had 75% of reduction in wear [42]. Darrith et al. [40] performed a systematic review involving 54 articles (10,783 primary THAs) in which either first-generation or modern DM cups were used. They did not find an intra-prosthetic dislocation in primary THAs performed after 2007.

### Stryker Trident acetabular shell

The Stryker Trident acetabular shell features titanium with grit-blasted hydroxyapatite coating, which has received the approval of the Food and Drug Administration (FDA) in 2003 [43]. The modular DM metal liner, made of cobalt chromium, engages the Trident shell in appropriate orientation of locking tabs and is impacted into the tapered shell [44]. Multiple studies examined the incomplete seating associated with the Trident ceramic metal-backed liner [45–48]. Similar complications due to incomplete seating of the modular DM liner were also reported recently. Padgett et al. [49] reported the incidence of mal-seated modular DM liner was 6% as shown by postoperative X-rays. In their series, 32 out of 521 Stryker acetabular cups were made on the basis of three different cup designs: Trident I hemispherical cup (8%), Trident I PSL cup (5%), and Trident II cup (4%). The clinical impact of mal-seated liner is still unknown. Theoretically, the risk of micromotion along the mal-seated interfaces leading to fretting and corrosion remains a concern.

### Tips for using modular DM system

Despite the improvements in modern DM, some tips are worth following for a successful THA. We put the tips into practice in the use the modular DM system, especially, to avoid the incomplete seating of the metal liner. First, excessive under-reaming of acetabulum should be avoided, because the impact caused by press-fitting may lead to in deformation of the metal acetabular shell [45–53]. This potentially causes mal-alignment of the cup and liner-locking mechanism, resulting in improper liner seating. The taper of the liner may also be damaged during the insertion, resulting in the failure of the taper-locking mechanism and seating failure. In a cadaveric study, Markel et al. [51] tested the press-fit technique using Trident acetabular shells, and found that compression deformation occurred in all their tests, with an average of 0.17 mm of pinch deformity. Second, the screw head should be completely countersunk into the screw hole in the acetabular cup, and avoid soft tissue interposition between the acetabular cup and modular DM liner. Third, the modular DM liner should be properly seated into the acetabular cup and checked radiologically as needed. Eskildsen et al. [54] reported that the inferomedial portion of the modular DM liner might be mal-seated even if the visible superior portion of modular DM seemed to be well-seated.

### Limitations of the study

The lacks of randomization and a comparison against non-DM bearing are the major limitations of the study. We did not do so because of our small sample size, insufficient statistical power, and various confounding factors that cannot be totally controlled. Longer-term follow-up is required to find out the actual incidence of late dislocation. The cups used in Asian populations are small, which may limit its utilization in Caucasian populations.

## Conclusions

In Asian populations with high risk of hip dislocation, the use of modular DM articulation produces promising outcomes, without causing hip dislocation, but the relatively small-sized acetabulum may limit its extensive application in other populations worldwide.

## Data Availability

All data analyzed during this study are included in this article.

## Abbreviations

DM: Dual Mobility
THA: Total Hip Arthroplasty
HHS: Harris Hip Score
